# The Mystery of Clopidogrel-Associated Inflammatory Arthritis

**DOI:** 10.7759/cureus.50564

**Published:** 2023-12-15

**Authors:** Faiza Javed, Shivam U Champaneria

**Affiliations:** 1 Hospital Medicine, University of Kentucky College of Medicine, Lexington, USA; 2 Oncology, University of Kentucky College of Medicine, Bowling Green, USA

**Keywords:** arthritis and orthopaedic rheumatology, oligo-arthritis, migratory arthritis, clopidogrel hypersensitivity, clopidogrel

## Abstract

Clopidogrel is an antiplatelet medication that plays an important role in the management and prevention of thrombotic vascular events in patients with acute coronary syndrome (ACS) and ischemic stroke. We report a case of a male patient who received a maintenance dose of clopidogrel as part of stroke treatment and developed inflammatory arthritis after five days of starting the medication. He underwent extensive evaluation and testing to explore other common causes of inflammatory arthritis, including autoimmune etiologies. None of the test results were helpful, and we hypothesized that his arthritis was induced by clopidogrel. Discontinuing this agent resulted in the complete resolution of the patient's symptoms. Since medication-induced arthritis is a diagnosis of exclusion, these patients should undergo a complete workup for inflammatory arthritis. If possible, a risk-benefit analysis of dual antiplatelet therapy (DAPT) in ischemic stroke patients with a prior history of rheumatoid arthritis (RA) should be done in collaboration with neurology.

## Introduction

Based on American Heart Association/American Stroke Association (AHA/ASA) guidelines, short-term dual antiplatelet therapy (DAPT) with aspirin and clopidogrel has become the standard of care in patients experiencing a qualifying transient ischemic attack (TIA) or minor ischemic stroke. We discuss a case of a male patient who was started on DAPT (aspirin and clopidogrel) based on these recommendations. However, within a few days of initiating the treatment, he developed inflammatory arthritis in multiple joints. We were not able to find any reports of such a presentation in any other patients who received DAPT (aspirin and clopidogrel) as a treatment for stroke [1,2].

## Case presentation

A 43-year-old male with a past medical history of ischemic stroke with no residual neurological deficits (diagnosed two weeks ago) presented with pain and redness of the right shoulder, right knee, left knee, and left third and fourth metacarpals for the last five days. The patient had been recently admitted for an ischemic stroke and discharged on aspirin, clopidogrel, and atorvastatin. Soon after discharge, he began to develop severe right shoulder pain associated with redness and tenderness, followed by similar symptoms in his right knee, left knee, and then right third metacarpal. The patient denied dysphagia, odynophagia, rashes, and any vision and neurologic changes and he had not had any unusual outdoor or travel exposures. There was no personal or family history of autoimmune disorders, rheumatic diseases, or inflammatory bowel disease.

**Figure 1:**
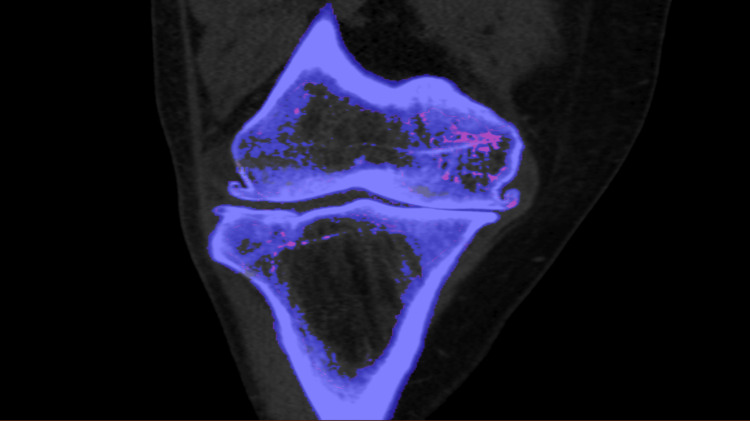
Dual-energy CT scan - right knee The image shows moderate-to-severe medial and mild lateral femorotibial compartment osteoarthrosis. There was no joint effusion, meniscal chondrocalcinosis, muscle or tendon atrophy, or crystals CT: computed tomography

Physical exam revealed swelling, erythema, and tenderness of the right shoulder and left knee. The cardiovascular system, abdomen, neurology, and skin examination were within normal limits. The initial laboratory showed normal cell counts, renal function, and liver function tests. Other notable labs included a serum uric acid level of 3.5 mg/dL and an erythrocyte sedimentation rate (ESR) of 35 mm/hr. Arthrocentesis of the left knee was performed, with a total nucleated cell count of 2000, polymorphonuclear neutrophils (PMNs) of 72%, no crystals, and negative Gram stain and culture. Further laboratory results included the absence of rheumatoid factor (RF) and lupus anticoagulant, negative anti-cyclic citrullinated (anti-CCP), antinuclear (ANA), antineutrophil cytoplasmic (ANCA), and anti-double-stranded DNA (anti-dsDNA) antibodies, and normal complements C3 and C4, proteins C and S, and serum IgE levels. ASO titer value was 189 IU/ml. Serum protein electrophoresis and immunofixation were unremarkable. Genetic testing for familial Mediterranean fever was also negative. Dual-energy CT (DECT) of the right and left knee showed moderate osteoarthritis with no crystals (Figure 1). The pain was initially relieved by Tylenol and topical capsaicin, but pain and tenderness persisted. Clopidogrel was stopped, and he was only continued on aspirin and atorvastatin for his ischemic stroke. Over the next three days, the patient's symptoms completely resolved, and the inflammatory markers returned to baseline. There was no recurrence of symptoms at his six-week follow-up visit with the primary care physician.

## Discussion

Polyarthritis refers to a joint disease that involves at least five joints. One or more signs of inflammation, including pain, movement restriction, swelling, warmth, and redness, are observed in the joints involved. If polyarthritis limits itself to less than six weeks, it is called acute polyarthritis. Our patient developed acute polyarthritis, given the involvement of the above-described joints, within one week of starting clopidogrel. The differential diagnoses we considered were autoimmune arthritis, infectious arthritis, reactive arthritis, gout, pseudogout, serum sickness disease, and atypical initial presentation of systemic lupus erythematosus (SLE), RA, or vasculitis. The workup for acute polyarthritis includes laboratory tests, imaging, and synovial fluid analysis. Laboratory workup includes complete blood count (CBC), liver function tests, urinalysis, acute phase reactants including ESR, CRP, serum amyloid A, complement components, alpha-1 antitrypsin, fibrinogen, ferritin, uric acid, creatine kinase, hepatitis panel, and lactate dehydrogenase (LDH). Autoantibodies are critical in diagnosing rheumatoid arthritis (RA), SLE, and systemic vasculitis, which can contribute to polyarthritis. These antibodies include RF, anti-CCP antibodies, and ANA. If any of these are positive, a complete autoimmune workup should be pursued. Synovial fluid analysis is also critical in the differential diagnosis of polyarthritis. Our patient initially presented with left knee swelling, necessitating synovial analysis. Lastly, imaging the joint spaces with plain radiographs may help visualize the disease process, although MRI is often ordered as it is more sensitive in differentiating inflammatory arthritic diseases.

Although our patient showed asymmetric joint involvement, he had no preceding gastrointestinal or urinary symptoms, fever, or rashes. Extraarticular manifestations such as dactylitis or uveitis were also absent. Inflammatory markers were normal to slightly elevated, making reactive, viral, or bacterial arthritis less likely. The absence of crystals in the synovial analysis does not completely rule out the possibility of crystal-induced arthropathy, and hence we performed a DECT scan.

Over the last two decades, there have been some reported cases of clopidogrel-induced migratory inflammatory polyarthritis [1-7], seen in patients receiving both loading and maintenance doses of clopidogrel as a part of the treatment for acute coronary syndrome (ACS). Our patient received 75 mg of clopidogrel as a treatment for acute stroke as per AHA/ASA guidelines. There were several common factors in these reported cases, such as the development of acute fevers, chills, and rashes followed by severe joint pain and inflammation seen approximately one week (on average) after the initiation of clopidogrel (Table 1).

**Table 1:** Compiled characteristics of patients with clopidogrel-induced migratory polyarthropathy P: pruritus; R: rash; F: fever; C: chills; ESR: erythrocyte sedimentation rate; CRP: C-reactive protein; WBC: white blood cell count; ALT: alanine transaminase; GGT: gamma-glutamyltransferase; ALP: alkaline phosphatase

Study	Patient age, years	Sex	Symptoms	Treatment indication	Onset following administration	Dosage	Joints involved	Laboratory values	Treatment
Kanadiya et al. [1]	52	Male	P (hands and feet), R, F, C	Progressive angina	2 weeks	75 mg qd	Bilateral knees, hips, shoulders, hands, and elbows	CRP: 171.7 mg/L, ESR: 68 mm/hr	Discontinuation, methylprednisolone
Agrawal et al. [2]	64	Male	F	Progressive angina, stent procedure	4 days	Not reported	Right shoulder, neck, bilateral wrists	CRP: 15 mg/dL, ESR: 89 mm/hr	Discontinuation, prasugrel, colchicine, indomethacin
Coulter and Montandon [3]	64	Male	C, F	Cardiac catheterization	8 days	75 mg qd	Left metacarpophalangeal joint, left talocrural, left ankle	CRP: 0.71 mg/dl, ESR: 26 mm/hr, WBC: 15.2 x 10^3^/mm^3^	Discontinuation, prasugrel
Bedy et al. [4]	65	Male	Swelling, redness, F, C	Pacemaker placement	2.5 weeks	75 mg qd	Hands, feet, elbow	CRP: 2.60 mg/dL, ESR: 35 mm/hr, WBC: 17.64 x 10^9^/L	Discontinuation, prednisone, ticagrelor
Chen et al. [5]	60	Female	F, P, rash (trunk, back)	gastroscopy	10 days	75 mg qd	Hips, shoulders, wrists, hands, knees	CRP: 408 mg/L, ESR: 64 mm/hr, ALT: 81 U/L, AST: 54 U/L, ALP: 157 U/L, GGT: 49 U/L	Discontinuation, prednisone, celecoxib
Khan et al. [6]	50	Male	Rash (limbs, trunk), F	Stent procedure	3 days	600 mg stat dose	Shoulders, right hand	CRP: 21 mg/L, urate: 0.45 mmol/l , GGT: 62 U/L, ALT: 43 U/L, eosinophils: 0.10 x 10^9^/L, WBC: 107,700 x 10^6^/L - left wrist aspiration	Discontinuation, ticlopidine
Ayesha et al., case 1 [7]	54	Male		Stent procedure	3 days	75 mg qd	Bilateral shoulders, bilateral hands, right hip	WBC: 13.5 K/mm^3^, ESR: 101-116 mm/hr, CRP: 29.7-26.6 mg/dL, uric acid: 10.9 mg/dl	Discontinuation, prasugrel
Ayesha et al., case 2 [7]	77	Male	F, C, weight loss, asthenia	Angina, percutaneous coronary intervention	2 weeks	75 mg qd	Left shoulder, left hip, right shoulder, left hand	ESR: 62-92 mm/hr, CRP: 13.9 15.3 mg/dL	Discontinuation, prasugrel

Our patient did not have any systemic symptoms such as fever, chills, or rashes. Similar to all other reported cases [1-7], we also observed the resolution of arthritis symptoms upon the discontinuation of clopidogrel. Clopidogrel-induced arthropathy is a diagnosis of exclusion. Clopidogrel is an older, safe medication, most commonly given to patients with ACS. Clopidogrel-induced arthritis is a rare entity [8]. Therefore, a comprehensive workup must be performed at the outset to rule out more common presentations such as lupus-associated arthropathy, statin-induced myalgias, or other autoimmune processes. The exact pathophysiology of clopidogrel-induced arthritis is currently unknown. However, a recent rat model study revealed that clopidogrel in combination with a chronic arthritis state could exacerbate arthritic-type symptoms. An increase in inflammatory cytokines such as IFN-y, IL-b, and IL-6 was also observed, which may have played a role in the previously reported cases [9]. Our patient developed the condition without any prior autoimmune disease or arthritis, and hence an even more thorough assessment of risks and benefits must be performed while using this medication in patients with a positive history. In some patients with inflammatory arthritis, it is not possible to establish a specific diagnosis during the first several weeks to months following symptom onset. However, the resolution of symptoms within three days of discontinuation of clopidogrel in our patient raised suspicion that the inflammatory response could be attributed to it. The patient was therefore followed up in six weeks to evaluate for any recurrence.

## Conclusions

Clopidogrel-induced arthritis is a rare entity and is a diagnosis of exclusion. Therefore, a comprehensive workup of the possible differential diagnoses should be performed in these patients. For some patients with inflammatory arthritis, it is not possible to establish a specific diagnosis in the first several weeks to months following symptom onset. All these patients should be closely followed up after discharge.

## References

[1] Kanadiya MK, Singhal S, Koshal VB (2011). Prasugrel as a safe alternative for clopidogrel-associated arthritis. J Invasive Cardiol.

[2] Agrawal S, Harburger J, Stallings G, Agrawal N, Garg J (2013). Clopidogrel-induced recurrent polyarthritis. J Investig Med High Impact Case Rep.

[3] Coulter CJ, Montandon SV (2012). Prasugrel as a safe alternative for clopidogrel-induced polyarthralgias. Pharmacotherapy.

[4] Bedy SC, Kesterson JP, Flaker G (2018). Ticagrelor as an alternative for clopidogrel-associated acute arthritis. Case Rep Emerg Med.

[5] Chen KK, Ginges I, Manolios N (2003). Clopidogrel-associated acute arthritis. Intern Med J.

[6] Khan EA, Blake JW, Stamp LK (2009). Ticlopidine as a safe alternative for clopidogrel-associated arthritis. J Rheumatol.

[7] Ayesha B, Varghese J, Stafford H (2019). Clopidogrel-associated migratory inflammatory polyarthritis. Am J Case Rep.

[8] Yusuf S, Zhao F, Mehta SR, Chrolavicius S, Tognoni G, Fox KK (2001). Effects of clopidogrel in addition to aspirin in patients with acute coronary syndromes without ST-segment elevation. N Engl J Med.

[9] Garcia AE, Mada SR, Rico MC, Dela Cadena RA, Kunapuli SP (2011). Clopidogrel, a P2Y12 receptor antagonist, potentiates the inflammatory response in a rat model of peptidoglycan polysaccharide-induced arthritis. PLoS One.

